# Predictors of toxicity for metastatic melanoma patients treated with ipilimumab

**DOI:** 10.1186/2051-1426-3-S2-P247

**Published:** 2015-11-04

**Authors:** Sara Valpione, Sandro Pasquali, Luca Campana, Simone Mocellin, Luisa Piccin, Jacopo Pigozzo, Vanna Chiarion-Sileni

**Affiliations:** 1 University of Padova, Padova, Italy; 2 IOV-IRCCS, Padova, Italy

## Introduction

Immunotherapy with ipilimumab, a monoclonal antibody anti-Cytotoxic T-Lymphocyte Antigen 4, demonstrated a survival advantage for metastatic melanoma patients, but the treatment is associated with immuno-related adverse events (irAEs) that may drive to severe comorbidities and challenge the treatment prosecution. No predictive factors for significant irAE (grade 3-4 according to Common Terminology Toxicity Criteria) are known. The purpose of this study was to identify prognostic factors for toxicity in melanoma patients treated with ipilimumab.

## Materials and methods

The following prospectively collected data were utilized: patient characteristics, previous therapies and level of serum biomarkers (LDH, C-reactive protein, β2-microglobulin, VEGF, IL2, IL6, S-100, ALP, transaminases, leukocyte count, lymphocytes subpopulations). Logistic regression was used for multivariate analysis of 140 consecutive metastatic patients treated with ipilimumab (3mg/kg, q3w) at Veneto Institute of Oncology (IOV). The study was approved by the local Ethic Committee and patients gave written informed consent.

## Results

Most patients (66%) completed the 4 cycles of therapy. Of the remaining patients, 24% withdrawn treatment for toxicity and 10% developed symptomatic central nervous system (CNS) metastases or rapid performance status (PS) worsening that required treatment interruption. Grade 3-4 diarrhea, which occurred in 19 patients (14%) was the most frequent cause of treatment discontinuation due to irAEs followed by hypophysitis, which occurred in 10 patients (7%). Patients experiencing G3-4 irAEs remained on corticosteroid therapy for a minimum of 4 weeks, to a maximum of 8 months of mineral-corticoid replacement in a case of hypophysitis (treatment ongoing).

**Figure 1:**
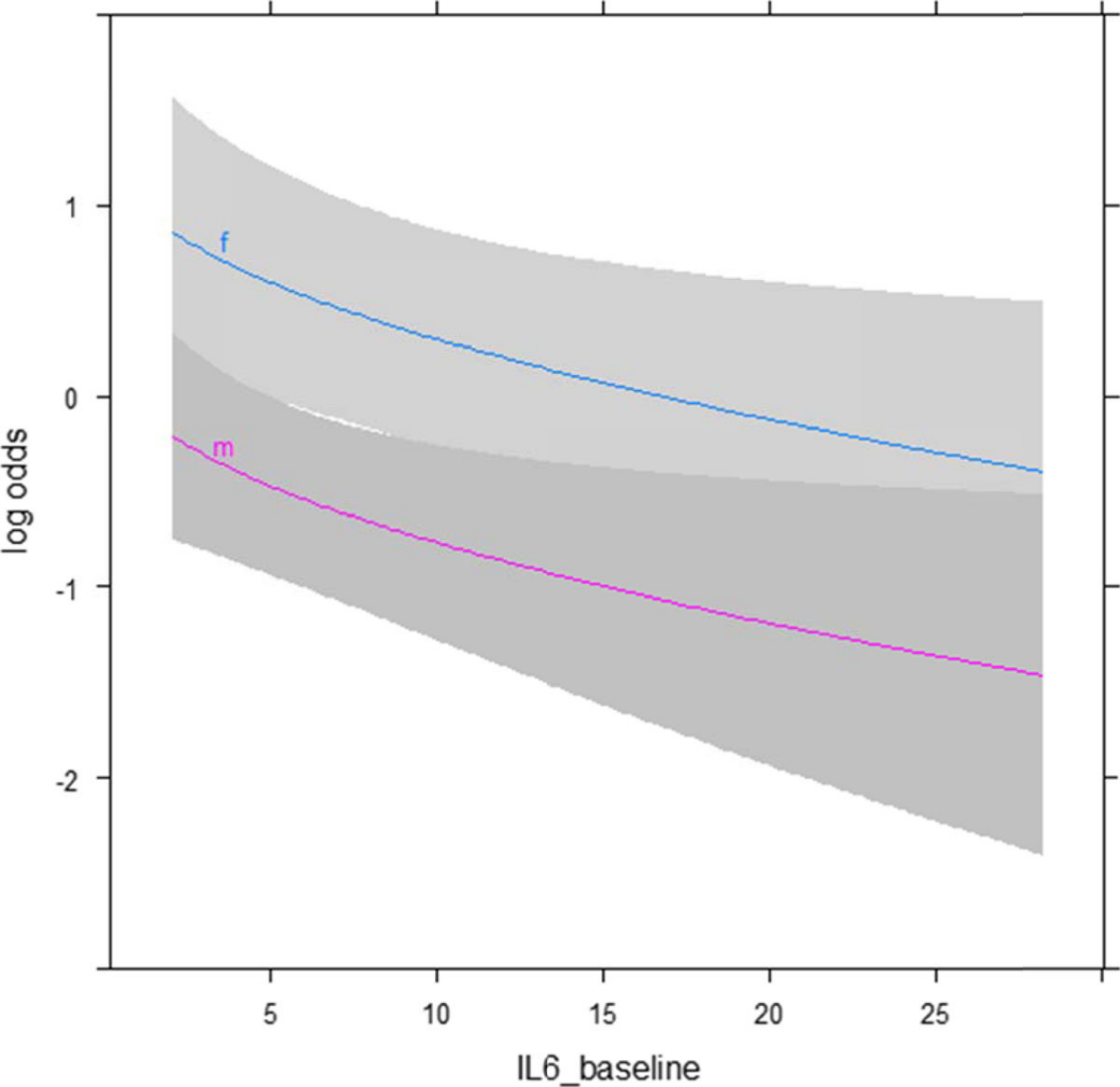


The association between collected clinical features and serum levels and grade 3-4 irAEs was investigated accounting for patients survival. Female patients and those with lower IL6 baseline serum levels were at higher risk of developing G3-4 toxicity (OR=1.5, 95% CI 1.06-2.16 and OR=2.84, 95% CI 1.34-6.03, respectively, Figure 1). These two variables were also the only significant at multivariate analysis (Chi-square 5.24, P=.022 and Chi-square 7.37, P=.007, respectively). No significant correlation with the subtype of irAE emerged from the cytokine analysis (P=.102). No organ-specific antibodies were detected.

## Discussion

Inflammatory environment may play a role in immune-tolerance and tumor response and it could also impact on immunorelated toxicities. The risk for autoimmune diseases is higher for female in the general population, for this reason our observations are not surprising. In conclusion, we propose to use baseline IL6 dosage before ipilimumab treatment, in particular in female, to identify patients at risk of toxicity, to monitor them up more carefully.

